# Machine Perfusion versus Cold Storage of Kidneys Derived from Donation after Cardiac Death: A Meta-Analysis

**DOI:** 10.1371/journal.pone.0056368

**Published:** 2013-03-11

**Authors:** Ronghai Deng, Guangxiang Gu, Dongping Wang, Qiang Tai, Linwei Wu, Weiqiang Ju, Xiaofeng Zhu, Zhiyong Guo, Xiaoshun He

**Affiliations:** Organ Transplant Center, the First Affiliated Hospital, Sun Yat-sen University, Guangzhou, China; University of Colorado, United States of America

## Abstract

**Background:**

In response to the increased organ shortage, organs derived from donation after cardiac death (DCD) donors are becoming an acceptable option once again for clinical use in transplantation. However, transplant outcomes in cases where DCD organs are used are not as favorable as those from donation after brain death or living donors. Different methods of organ preservation are a key factor that may influence the outcomes of DCD kidney transplantation.

**Methods:**

We compared the transplant outcomes in patients receiving DCD kidneys preserved by machine perfusion (MP) or by static cold storage (CS) preservation by conducting a meta-analysis. The MEDLINE, EMBASE and Cochrane Library databases were searched. All studies reporting outcomes for MP versus CS preserved DCD kidneys were further considered for inclusion in this meta-analysis. Odds ratios and 95% confidence intervals (CI) were calculated to compare the pooled data between groups that were transplanted with kidneys that were preserved by MP or CS.

**Results:**

Four prospective, randomized, controlled trials, involving 175 MP and 176 CS preserved DCD kidney transplant recipients, were included. MP preserved DCD kidney transplant recipients had a decreased incidence of delayed graft function (DGF) with an odd ration of 0.56 (95% CI = 0.36–0.86, P = 0.008) compared to CS. However, no significant differences were seen between the two technologies in incidence of primary non-function, one year graft survival, or one year patient survival.

**Conclusions:**

MP preservation of DCD kidneys is superior to CS in terms of reducing DGF rate post-transplant. However, primary non-function, one year graft survival, and one year patient survival were not affected by the use of MP or CS for preservation.

## Introduction

In order to address the severe shortage of organs available for clinical transplantation, donation after cardiac death (DCD) donors have reemerged as an additional source of organs in many countries during the last decade [1]. Unlike kidneys from donation after brain death (DBD), DCD kidneys are subjected to a substantial period of warm ischemic injury (time from cardiac arrest to perfusion with cold preservation solution). As a result, DCD kidneys suffer from a higher incidence of delayed graft function (DGF) than kidneys from DBD donors (28–88% vs. 13–35%) [1], [2], [3]. Since DGF is associated with a higher risk of acute rejection, longer hospital stay, and poorer long-term graft outcomes [4], [5], reducing the incidence of DGF is currently a major issue in kidney transplantation.

Since the time that kidney transplantation was first implemented as a clinical standard of care, two preservation technologies have been developed to minimize ischemic injury to kidney allografts prior to transplantation, namely static cold storage (CS) and pulsatile machine perfusion (MP) [6]. The use of MP declined dramatically after the introduction of the University of Wisconsin (UW) solution, which could preserve DBD kidneys for up to 24 hours by simple cold storage [6]. However, with the increasing use of DCD donors over the past decade, the MP system has regained attention as an important option for preservation. DCD kidneys are associated with a higher DGF rate than those from DBD donors due to a longer warm ischemic period which is unavoidable in the process of organ procurement from DCD donors. Optimizing the preservation of such kidneys has become a significant issue in the field. It has been suggested that MP preservation of DCD kidneys results in a lower rate of DGF and better graft survival compared with those preserved by CS [7], [8], [9]. A meta-analysis of machine perfusion in 2003 concluded that MP is superior to CS in terms of DGF for deceased donor (DD) kidneys, although the one year graft survival rate is comparable [10]. Subsequently, a multicenter, randomized, controlled trial (RCT) in 2009 with a majority of DBD donors also suggests that MP is superior to CS for DD donor kidney transplants [11]. Recently, however, two well-powered multicenter RCTs demonstrated controversial outcomes for DCD kidneys [12], [13]. Of the two RCTs, the one performed in Belgium and the Netherlands indicated that MP reduced the incidence of DGF from 69.5% to 53.7%, whereas the other one carried out in the United Kingdom found no difference in the incidence of DGF between kidneys preserved by MP or CS (58% vs. 56%, respectively).

Considering the current controversy, we conducted a meta-analysis of the available relevant prospective RCTs to better understand whether MP is able to gain better outcomes in DCD kidney transplantation when compared to CS. These data convey an important message for clinical transplant professionals to decide the best way to preserve DCD kidneys regarding the transplant outcomes.

## Materials and Methods

### Data sources and searches

A search of the PubMed/Medline, Embase and Cochrane library databases was performed using the terms (“MP” OR “machine perfusion” OR “machine preservation” OR “extracorporeal perfusion”) AND (“DCD” OR “donation after cardiac death” OR “NHBD” OR “non-heart-beating donors”). The search was conducted in May 2012. Publications were limited to those written in English and to those reporting results from human subjects. Review articles were excluded after limit filtering. A manual search of the references of the relevant publications was also performed.

### Study selection: Inclusion and exclusion criteria

Studies reporting outcomes of DCD kidney transplantation using MP preservation were included. Exclusion criteria were: (1) Overlapping cohort studies from the same institution (avoid duplication). (2) Studies lacking a control group (CS preservation). (3) Studies that included DBD donors. (4) Retrospective, non-randomized, or uncontrolled design studies. (5) Studies published greater than 20 years ago were excluded due to the significant technological changes that occurred in preservation techniques and other advances in transplantation surgery after that time [14]. (6) Finally, given that the outcomes of kidney grafts depend strongly on immunotherapy, we considered it inappropriate to include studies applying different immunosuppressant regimens within the two groups in the same cohort study, especially for the induction therapy.

### Quality assessment and data extraction

Publications were reviewed and data were extracted by two independent investigators with disagreements being resolved through discussion and consensus. The primary outcome was the incidence of DGF, defined as the requirement of dialysis in the first week after transplantation. Secondary outcomes included primary non-function (PNF), one year graft survival, and one year patient survival rates.

### Data Synthesis and Analysis

Pooled odds ratios (OR) were used to evaluate the event rates, and the results were reported with 95% confidence intervals (CI). A P value <0.05 was considered a significant difference in the values between the two groups. Heterogeneity through all the included studies was evaluated by χ^2^ and *I^2^* statistical tests. Heterogeneity was considered significant when P<0.05 or *I^2^*>50%, and a random effect model was adopted. A random effect model is a kind of hierarchical linear model which assumes that the data set being analyzed consists of a hierarchy of different populations whose differences relate to that hierarchy. When P>0.05 for χ^2^ or *I^2^*<50% for *I^2^* statistical tests, indicating low statistical heterogeneity in both cases, a fixed effect model was used. A fixed effect model is a statistical model that represents the observed quantities in terms of explanatory variables that are treated as if the quantities were non-random. Publication bias was assessed by a funnel plot. A funnel plot is designed to check the existence of publication bias in systematic reviews and meta-analyses. The largest studies will be near the average while small studies will be spread on both sides of the average. Variation can indicate publication bias. All statistical analyses for the current study were performed with Review Manager (RevMan Version 5.1, 2008. The Nordic Cochrane Centre, Rigshospitalet).

## Results

### Search results and included studies

**Figure 1:**
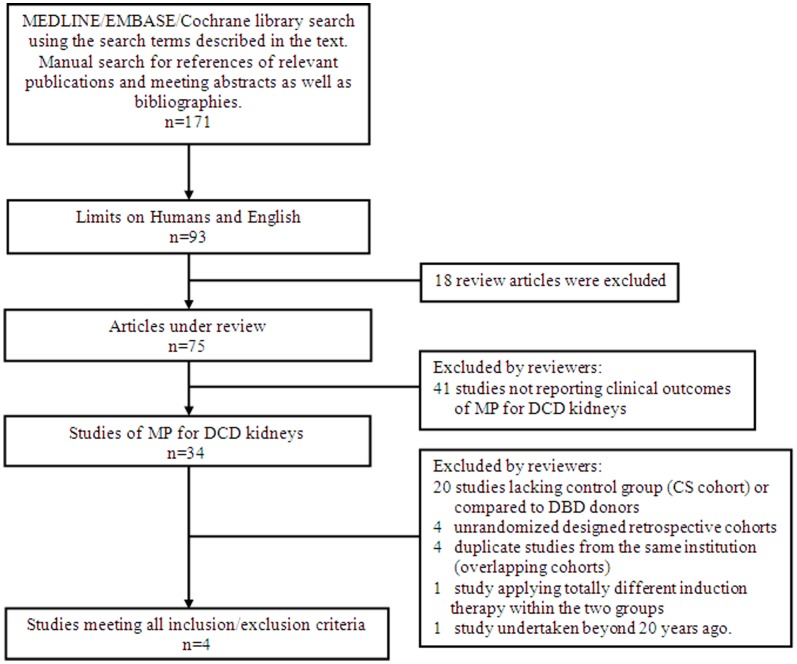
Search algorithm and study selection outcomes.

The search algorithm and results based on the search strategies and selection criteria described above are outlined in Figure 1. Briefly, 171 articles were initially identified. Seventy-five studies remained after excluding animal studies, review articles and non-English reports. Among those references, 41 studies not reporting clinical outcomes of MP for DCD kidneys were excluded. The remaining 34 publications of MP for DCD kidneys underwent extensive review. Nineteen of these studies lacked a control (CS cohort) group or compared the results of MP organs from DCD donors to CS organs from DBD donors and were excluded from this meta-analysis. Four duplicate studies from the same institution (overlapping cohorts), four non-randomized designed retrospective studies, one study [15] applying different induction therapy within the two groups, as well as one study [16] performed in 1977 (more than 20 years ago) were also excluded. Two meeting abstracts were identified by manual search and both were retrospective cohorts and were not included in this study. Finally, four studies meeting all criteria were included and the study characteristics are shown in Table 1. No evidence of publication bias among the included studies was found by means of a funnel plot. One hundred seventy-five patients receiving MP DCD kidneys and 176 recipients receiving CS DCD kidneys were included in this meta-analysis. Three of the studies were from European institutes, while the remaining one was from Asia (Japan). Table 2 shows the primary and secondary outcomes and MP details of each included study.

**Table 1:** Characteristics of the included studies.

					Population Demographics					
		Sample Size				Recipient Age		CIT (hours)Mean (range)		WIT (min)Mean (range)
References	Institute	MP	CS	Study periods	Donor Age	MP	CS	MP	CS	
Ina Jochmans (2010) [12]	Eurotransplant Multicenter	82	82	2005–2007	43(17–67)	49(24–73)	52(24–77)	15.0(4.3–28.9)	15.9(8.6–46.6)	16(6–38)
Watson (2010) [13]	United Kingdom Multicenter	45	45	2006–2007	45.6±14.6	50.3±14.2	48.6±13.9	13.9(6.7–24.2)	14.3(7.0–30.1)	15(4–35)
van der Vliet (2001) [17]	The Netherlands Multicenter	35	36	N/A	36.6±2.7	N/A	N/A	25.0±1.0	23.0±1.3	28.4 (4–47)
Matsuno (1994) [18]	Japan	13	13	N/A	50.1	38.5±10.1	41.0±7.9	11.9±6.2	6.08±2.93	0
	Totals:	175	176							

MP, Machine Perfusion; CS, Static Cold Storage; CIT, Cold Ischemic Time; WIT, Warm Ischemic Time; N/A, non-available.

**Table 2:** Outcomes and MP details of each included study.

References	Type of MP/Perfusate	Primary outcome			Secondary outcomes								
		DGF			PNF			1 yr graft survival			1 yr patient survival		
		MP(%)	CS(%)	*p*	MP(%)	CS(%)	*p*	MP(%)	CS(%)	*p*	MP(%)	CS(%)	*p*
Ina Jochmans (2010) [12]	LifePort/KPS-1	53.7	69.5	0.007	2.4	2.4	1.00	93.9	95.1	N/A	96.3	97.6	N/A
Watson (2010) [13]	LifePort/KPS-1	57.8	55.6	0.99	2.22	0.00	N/A	93.3	98.0	0.3	93%	100%	0.08
van der Vliet(2001) [17]	Gambro/Belzer	40.0	66.7	0.15	17.1	11.1	0.15	76.3	84.2	N/A	N/A	N/A	N/A
Matsuno (1994) [18]	APS-02/cryoprecipitated plasma	61.5	84.6	<0.05	0.00	7.60	N/A	N/A	N/A	N/A	N/A	N/A	N/A

MP, Machine Perfusion; CS, Static Cold Storage; DGF, Delayed Graft Function; PNF, Primary Non-function; N/A, non-available.

### Primary outcome

**Figure 2:**
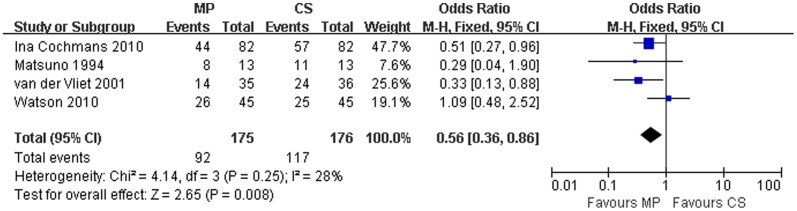
DGF rates for DCD kidneys preserved by MP versus CS. Pooled estimate of odds ratio (OR) and 95% confidence intervals (CIs) of DGF rates for DCD kidneys preserved by MP on 5 studies. Squares indicate OR in each study. The square size is proportional to the weight of the corresponding study; the length of horizontal lines represents the 95% CI. The rhombus indicates the pooled OR and 95% CI (fixed-effect model).

All four studies reported the incidence of DGF in MP and CS transplants, and while two of the reports demonstrated that MP reduced the incidence of DGF compared to CS, the other two publications did not show a significant difference in outcomes. The overall rate of DGF was 52.6% (range: 40%–61.5%) for MP and 66.5% (55.6%–84.6%) for CS kidneys. There was no significant heterogeneity among the 4 studies (χ^2^ = 4.14, P = 0.25; *I* ^2^ = 28%). Thus, a fixed effect model was used. Combined result of the 4 studies showed that the OR for DGF was 0.56 (95% CI = 0.36–0.86) for MP over CS (P = 0.008) (Figure 2).

### Secondary outcomes

**Figure 3:**
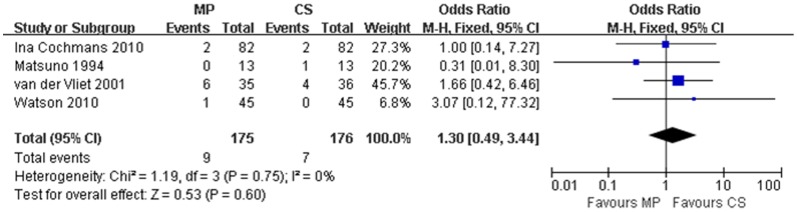
PNF rates for DCD kidneys preserved by MP versus CS. Pooled estimate of odds ratio (OR) and 95% confidence intervals (CIs) of PNF rates for DCD kidneys preserved by MP on 4 studies. Squares indicate OR in each study. The square size is proportional to the weight of the corresponding study; the length of horizontal lines represents the 95% CI. The rhombus indicates the pooled OR and 95% CI (fixed-effect model).

All four [12], [13], [17], [18] studies reported primary non-function (PNF) rates post-transplantation. Recipients receiving MP or CS kidneys from DCD donors experienced similar PNF incidence, with overall rate of 5.1% or 4.0%, respectively. No heterogeneity was identified through the 4 studies (χ^2^ = 1.19, P = 0.75; *I* ^2^ = 0%), thus a fixed effect model was adopted. The OR for developing PNF in transplant recipients receiving MP preserved kidneys was 1.30 (95% CI = 0.49–3.44) compared to those receiving CS preserved kidneys, which was not statistically significant (P = 0.60) (Figure 3).

**Figure 4:**
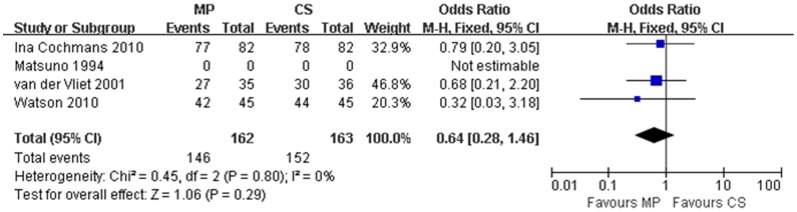
One year graft survival for DCD kidneys preserved by MP versus CS. Pooled estimate of odds ratio (OR) and 95% confidence intervals (CIs) of one year graft survival for DCD kidneys preserved by MP on 4 studies. Squares indicate OR in each study. The square size is proportional to the weight of the corresponding study; the length of horizontal lines represents the 95% CI. The rhombus indicates the pooled OR and 95% CI (fixed-effect model).

We further evaluate the graft and patient survival at one year after transplantation in order to understand whether the reduction of DGF rate from MP preservation could result in better one year graft and patient survival. Of all four studies, three [12], [13], [17] reported one year graft survival rates while only two [12], [13] reported patient survival rates at this time point. There was no significant heterogeneity among the three studies that reported one year graft survival rates (χ^2^ = 0.45, P = 0.80; *I* ^2^ = 0%) Similarly, no significant heterogeneity was observed between the two studies that reported one year patient survival rates (χ^2^ = 0.83, P = 0.36; *I* ^2^ = 0%). Thus, a fixed effect model was used to pool the overall results for one year graft survival rate and one year patient survival rate. For MP preservation, the OR for one year graft survival was 0.64 (95% CI = 0.28–1.46), and there was no significant difference between the two preservation techniques (P = 0.29) (Figure 4). For one year patient survival, the odds ratio for MP preservation was 0.37 (95% CI = 0.09–1.64) compared to CS preservation; this value was not statistically significant (P = 0.19).

We attempted to evaluate the length of stay (LOS), mean duration of dialysis for DGF period and serum creatinine levels at 3 months post-transplantation. However, only one study [12] provided LOS data, and there was no difference between the two methods. Two studies reported mean duration of dialysis [13], [18] and serum creatinine levels at 3 months [12], [17] after transplantation, but the published data was not sufficient for combined analysis.

## Discussion

Because of the increasing number of patients added to the transplant waiting list and the lack of sufficient standard criteria donors (SCD) (brain death donors less than 50 years old) to meet this demand, DCD kidneys are now being accepted by many organ transplant centers all over the world as a potential organ source [19]. Previous clinical studies showed that this kind of kidney had a higher risk of complications such as DGF when compared to SCD kidneys. Some studies demonstrated that MP preservation could reduce DGF rate in both SCD and DCD kidneys. In 2003, a meta-analysis showed that MP led to a 20% reduction in the incidence of DGF [10]. However, there was considerable clinical heterogeneity among the involved studies, which included prospective RCTs with small sample size and even retrospective non-RCTs. This conclusion was thought to be premature to advocate the widespread use of MP into clinical kidney transplantation [10]. Thereafter, two multicenter RCTs were conducted in Europe and controversial conclusions were drawn. Our analysis, based on prospective RCTs of DCD kidneys, identified that MP is superior to CS in lowering the incidence of DGF in DCD kidneys, with a ∼40% reduction in DGF when organs are preserved by MP compared to those preserved by CS. We found no prospective RCT study conducted in the US using our searching strategy. In 2006, data from Scientific Registry of Transplant Recipients (SRTR) concerning the use of DCD organs showed that the rates of DGF were not significantly different between MP and CS for DCD kidneys (40.2% vs. 42.3%, P = 0.15) (data from 2000 to 2004) [20]. A meeting abstract using the OPTN/UNOS database (from 2004 to 2009) also found that there was no difference in DGF rate between MP and CS (43.1% vs. 42.3%, P = 0.83) for DCD kidneys. However, another publication based on OPTN data (from 1993 to November 2008) analyzing DCD donors found that MP did reduce DGF incidence compared with CS when donor age was >60 years and improved long term graft survival when donor age was >50 years [21]. Importantly, the ratio of controlled to uncontrolled DCD is higher in the US than in Europe, and the incidence of DGF is lower in the US than in Europe (∼40% vs. ∼60%) [12], [13]. Overall, although no subgroup analysis was done in the present study, we believe MP benefits early outcome (DGF) of DCD kidneys, especially when the organs are from expanded-criteria donors (ECD) (brain death donors more than 60 years old or between 50 and 59 years of age with 2 of the 3 additional risk factors) and uncontrolled DCD donors.

We found no difference in the incidence of PNF between the two preservation methods in the current analysis. This may be true or may be due to an inability to detect an effect of MP because of the relatively low incidence of PNF (∼5%) after DCD kidney transplant. One would expect that the reduction of DGF by machine perfusion might lead to an increase in graft and patient survivals since previous studies suggested that DGF was associated with reduced graft survival, which was observed in DBD kidneys. However, in our study, the one year graft and patient survival rates were similar between MP and CS preserved DCD kidneys. One US study [21] also revealed the same results based on a retrospective cohort of the OPTN/UNOS database (from 2004 to 2009) (The mean follow-up time was 2.2±2.6 years with a range of 0–15 years). The different nature of DGF between DCD and DBD kidneys may be one of the underlying reasons for this observation. Metabolic, hemodynamic, hormonal, and inflammatory changes triggered by brain death [22], but not cardiac death may impair kidney function more than warm ischemic injury alone, thus affecting long-term outcomes of DBD kidneys more [23]. Though MP was not shown to benefit one year graft and patient survivals based on the current study, it may increase long-term graft and patient survivals because of the benefit of reducing DGF incidence since DGF has been correlated with decreased long-term graft survival [21]. Indeed, one recent multicenter RCT found that the 3-year DBD kidneys allograft survival preserved by MP is better than those with CS preservation, especially in ECD kidneys [24].

Notably, MP needs additional logistic requirements and costs in comparison with conventional CS preservation before the kidneys are implanted. However, reduced requirement for dialysis after transplantation due to a lower rate of DGF and a shorter duration of DGF after transplantation could compensate for these additional logistic requirement costs by MP. No included study assessed the cost-effectiveness of MP and CS for DD kidneys in this analysis, but one multicenter RCT study [25] evaluated the short-term cost-effectiveness and showed that total initial hospitalization cost is less for MP than for CS, suggesting that MP can lower the costs per life-year and reduce costs per quality-adjusted life-year (QALY) when compared to CS.

There are some limitations in the present study. Firstly, clinical heterogeneity between studies might exist although we had strict enrollment criteria of references (only included prospective RCTs). One of the studies [16], [18] used the cryoprecipitated plasma perfusate for MP while the two most recent studies used the kidney preservation solution-1 (KPS-1) [12], [13]. In addition, the current analysis only presented data about the short term patient and graft survival, MP likely affect the long term patient and graft survival. Furthermore, this study included all four kinds of DCD donors (Maastricht classification: I: Brought in dead; II: Unsuccessful resuscitation; III: Awaiting cardiac arrest in hospital; IV: Cardiac arrest after brain-stem death) though category III DCD donor was the main portion of the study population and the only type of subject in the two multicenter RCTs [12], [13]. Thus, both controlled and uncontrolled cardiac death donors were pooled together in our analysis. This may result in insufficient evidence to determine whether this result is suitable for controlled or uncontrolled DCD donors only. Additionally, though we included studies no more than 20 years old, clinical practice of MP during this period may differ between studies as stated in the Results section. Finally, none of the included studies performed a cost-effectiveness analysis. These limitations provide room for future studies.

In conclusion, MP preservation of kidneys obtained from DCD donors can reduce DGF incidence after transplantation in comparison to conventional CS preservation. However, PNF incidence and one year graft and patient survivals were not different in patients using the two technologies. MP preservation is recommended for DCD kidneys to minimize the risk of DGF.

## Supporting Information

PRISMA Checklist S1 PRISMA 2009 Checklist.
(DOC)

Flow Diagram S1 PRISMA 2009 Flow Diagram.
(DOC)
