# Supplementary material for: Machine Perfusion versus Cold Storage of Kidneys Derived from Donation after Cardiac Death: A Meta-Analysis
Source: PLoS One. 2013 Mar 11;8(3):e56368. doi: 10.1371/journal.pone.0056368 (PMC3594243; doi:10.1371/journal.pone.0056368)
Supplement: Flow Diagram S1 — PRISMA 2009 Flow Diagram. (DOC) [file pone.0056368.s002.doc]

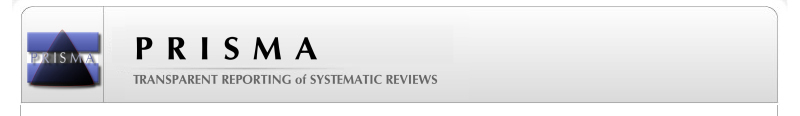
**PRISMA 2009 Flow Diagram**

**Identification**

MEDLINE/EMBASE/Cochrane library search using the search terms described in the text. Manual search for references of relevant publications and meeting abstracts as well as bibliographies.

n=171

**Screening**

Limits on Humans and English

n=93

18 review articles were excluded

**Eligibility**

Articles under review

n=75

Excluded by reviewers:

41 studies not reporting clinical outcomes

of MP for DCD kidneys

Studies of MP for DCD kidneys

n=34

Excluded by reviewers:

20 studies lacking control group (CS cohort) or

compared to DBD donors

4 unrandomized designed retrospective cohorts

4 duplicate studies from the same institution

(overlapping cohorts)

1 study applying totally different induction therapy within the two groups

1 study undertaken beyond 20 years ago.

**Included**

Studies meeting all inclusion/exclusion criteria

n=4
